# Multidisciplinary tumor board analysis: validation study of a central tool in tumor centers

**DOI:** 10.1007/s00277-022-05051-y

**Published:** 2022-12-05

**Authors:** Benedikt Frank, Gabriele Ihorst, Georg Herget, Henning Schäfer, Jakob Neubauer, Marc-Antoine Calba, Daniel Textor, Mandy-Deborah Möller, Sina Wenger, Johannes Jung, Johannes Waldschmidt, Cornelius Miething, Michael Rassner, Christine Greil, Ralph Wäsch, Monika Engelhardt

**Affiliations:** 1 Department of Internal Medicine I, Focus On Hematology, Oncology and Stem Cell Transplantation, University Medical Center Freiburg, Faculty of Medicine, University of Freiburg, Hugstetterstraße 53, 79106 Freiburg, Baden-Württemberg Germany; 2 Clinical Trials Unit, Faculty of Medicine, University of Freiburg, Freiburg, Germany; 3 Tumor Center Freiburg, Comprehensive Cancer Center Freiburg (CCCF), Freiburg, Germany; 4 Clinic for Orthopedics and Trauma Surgery, Department of Surgery, Medical Faculty, University of Freiburg, Freiburg, Germany; 5 Clinic for Radiotherapeutics, University of Freiburg, Freiburg, Germany; 6 Department of Radiology, University of Freiburg, Freiburg, Germany; 7 Pathology, University of Freiburg, Freiburg, Germany

**Keywords:** Multiple myeloma (MM), Tumor boards (TBs), Validation assessment, Left-truncated survival times

## Abstract

The established standard to ensure state-of-the-art cancer treatment is through multidisciplinary tumor boards (TBs), although resource- and time-intensive. In this validation study, the multiple myeloma (MM)-TB was reexamined, aiming to validate our previous (2012–2014) results, now using the TB data from March 2020 to February 2021. We assessed MM-TB protocols, physicians’ documentation, patient, disease, remission status, progression-free survival (PFS), and overall survival (OS) as left-truncated survival times. Moreover, TB-adherence, level of evidence according to grade criteria, time requirements, study inclusion rates, and referral satisfaction were determined. Within a 1-year period, 312 discussed patients were documented in 439 TB protocols. Patient and disease characteristics were typical for comprehensive cancer centers. The percentages of patients discussed at initial diagnosis (ID), with disease recurrence or in need of interdisciplinary advice, were 39%, 28%, and 33%, respectively. Reasons for the MM-TB presentation were therapeutic challenges in 80% or staging/ID-defining questions in 20%. The numbers of presentations were mostly one in 73%, two in 20%, and three or more in 7%. The TB adherence rate was 93%. Reasons for non-adherence were related to patients’ decisions or challenging inclusion criteria for clinical trials. Additionally, we demonstrate that with the initiation of TBs, that the number of interdisciplinarily discussed patients increased, that TB-questions involve advice on the best treatment, and that levels of compliance and evidence can be as high as ≥ 90%. Advantages of TBs are that they may also improve patients’, referrers’, and physicians’ satisfaction, inclusion into clinical trials, and advance interdisciplinary projects, thereby encouraging cancer specialists to engage in them.

**Supplementary Information:**

The online version contains supplementary material available at 10.1007/s00277-022-05051-y.

## Introduction

The established standard to ensure state-of-the-art cancer treatment — as a prerequisite in the national cancer plan and for comprehensive cancer centers (CCCs) — is through multidisciplinary tumor boards (TBs). Since this is compulsory for outstanding cancer centers, different analyses on TBs have been performed, because these are resource-, personnel-, and time-intensive [1]. Performed outcome measures have been better cancer care through interdisciplinary teams [2–5], improved response, progression-free survival (PFS) and overall survival (OS), enhanced adherence through electronically available TB protocols, higher patients’ and physicians’ satisfaction with cancer care [1, 6], and easier patient inclusion into clinical trials [1].

Since treatment options for tumor diseases, including multiple myeloma (MM), have improved considerably in recent years [7], cancer care has become more complex due to numerous therapeutic options. In addition, MM patients, with symptomatic disease and SLIM-CRAB criteria (≥ 60% clonal bone marrow plasma cells, involved/uninvolved free light chain (FLC) ratio of ≥ 100, MRI with > 1 focal lesion, hypercalcemia, renal insufficiency, anemia, osteolytic bone lesions), are often treated by different disciplines and require multidisciplinary care to coordinate complex therapeutic options [1, 8–10]. TBs represent an established way to meet this goal [1, 8–10]. Notably, comprehensive TB analyses of other tumor entities have demonstrated efficient treatment decisions and adjustments of initial treatment plans in ~ 30% [2, 3, 11–17]. In our initial analysis using our MM-TB and data from 2012–2014, we had described (a) what clinical questions were solved, (b) the level of adherence and evidence for decisions, (c) the frequency of participation in clinical trials, and (d) the level of satisfaction among participants, referrers, and patients [1].

In our MM-TB, five disciplines, namely hematologists/oncologists, radiologists, orthopedists, radiation therapists, and pathologists, are obligatorily attending each week, while physicians and experts of other disciplines (i.e., nephrologists, neurologists, cardiologists) are included according to pending clinical questions. Due to the prolonged SARS-CoV-2 pandemic [18, 19], electronic access to all 26 University of Freiburg (UKF)-TBs has been ensured, which allows disciplines with frequent involvement to resourcefully participate therein. In this analysis, the interdisciplinary approach to MM-TB was examined with the inclusion of hematologists/oncologists, radiologists, orthopedic surgeons, radiation therapists, pathologists, referring physicians, and primary care physicians. This study aimed to validate the results of our prior analysis [1] using the MM-TB data from March 2020 to February 2021.

## Methods

### Data analysis

For the analysis, all MM-TB protocols, physician letters, physician/nurse documentation, and electronic tumor baseline documentation over a 12-month period (March 2020–February 2021) were analyzed and compared with our preliminary analysis from 2012 to 2014 [1, 9], whenever appropriate (Table 1). We performed this exploratory study in 312 consecutive MM patients, discussed in 439 MM-TB protocols, who received in- or outpatient care at our CCCF. Patient characteristics included age, gender, Karnofsky performance status (KPS), revised myeloma comorbidity index (R-MCI), and Charlson Comorbidity Index (CCI). Via R-MCI and CCI scores, all patients were divided into fit, intermediate fit, or frail (Table 1). Myeloma-specific data were determined (including precursor disease stages monoclonal gammopathy of undetermined significance [MGUS] or smouldering multiple myeloma [SMM]), paraprotein types, International Staging System (ISS), revised-ISS (R-ISS), CRAB criteria, and remission status at the time of the MM-TB discussion) (Table 2). Quality of response was assessed via IMWG (International Myeloma Working Group) criteria.

**Table 1:** Patient and myeloma characteristics

	Current study 2020–2021 (*n* = 312 pts; *n* = 439 TB-protocols)	*Prior study Engelhardt 2012–2014 (n* = *299 pts; n* = *498 TB-protocols)*	Comparison 2020/2021:2012–2014
Evaluation period	March 2020–February 2021	*June 2012–June 2014*	
Median # TB protocols/pt_during evaluation period_	1 (1–10)	*2 (1–8)*	Similar number of pts, but 2020/2021 less TB protocolls / pt (see ^1^ and ^2^ for details)
*Patient-specific data*			
Median age (range)^3^	67 (35–94)	*67 (10*^*4*^*–89)*	Comparable
Males:females	56%:44%	*56%:44%*	Comparable
Median # comorbidities (range)^4^	5 (0–19)	*5 (0–19)*	Comparable
Median KPS (%; range)^4^	80 (50–100)	*80 (10–100)*	Comparable
Median R-MCI (range)^4^	4 (0–9)	*n.a*	
Median CCI (range)^4^	2 (0–10)	*n.a*	
*Myeloma-specific data*			
MM/MGUS/Others (SMM, M.Waldenström)	74%/12%/14%	*77%/8%/15%*	Comparable
IgG/IgA/IgM/IgD/asecretory/biclonal/LC	53%/16%/3%/1%/1%/2%/24%	*53%/17%/4%/0,3%/0,7%/1%/21%*^*#*^	Comparable
Κ:λ/asecretory/biclonal	63%:34%/1%/2%	*63%:32%/0.7%/0.3% *^*#*^	Comparable
ISS: I/II/III	48%/25%/27%	*45%/24%/31%*	Comparable
R-ISS: I/II/III	31%/48%/21%	*n.a*	
CRAB^1^: C/R/A/B # CRAB^4^: 0/1/2/3/4	2%/16%/23%/63% 29%/46%/19%/5%/1%	*n.a*	
Remission status^4^:			Current cohort with slightly better remission
ID/PD/ ≥ SD/diagnosis finding	39%/28%/33%/-	*34%/36%/26% /4%*	

*n* sample size, *n.a.* not assessed, *pt/pts* patient/patients, *TB* tumor board, *#* number, *range* min.–max. distribution, *KPS* Karnofsky Performance Status, *R-MCI* Myeloma-specific Comorbidity Index, see www.myelomacomorbidityindex.com, *CCI* Charlson Comorbidity Index,—not assessed in prior analysis‚ *MM* multiple myeloma, *MGUS* monoclonal gammopathy of uncertain significance, *SMM* smoldering MM, *LC-MM* light-chain MM, *k* kappa, *l* lambda, *(R-)ISS* (revised) international staging system, *CRAB* hyerpcalcema, renal impairment, anemia, bone lesions, *ID* initial diagnosis, *PD* progressive disease, *SD* stable disease

^*#*^ In 3% and 4% missing heavy chain information in prior analysis of externally presented patients

^1 ^Engelhardt M. et al. Multidisciplinary Tumor Boards: Facts and Satisfaction Analysis of an Indispensable Comprehensive Cancer Center Instrument. Dtsch Med Wochenschr. 2017 May;142(9):e51-e60

^2 ^Engelhardt M. et al. Multidisciplinary Tumor Boards and their analyses: the yin and yang of outcome measures. BMC cancer 2021;21:173

^3 ^Ten-year-old boy with University of Freiburg- and reference-histologically confirmed plasmocytoma in the nasopharynx, in sustained remission after local therapy

^4 ^At the time of TB presentation

**Table 2:** Therapy- and TB-specific data

	2020–2021 (*n* = 312 pts; *n* = 439 TB-protocols)	*Prior study 2012–2014 (n* = *299 pts; n* = *498 TB-protocols)*	Comparison 2020/2021:2012–2014
*Therapy-specific data*			
Prior therapies^1^:	23%/30%/41%/6%		
Standard ± NA/ASCT + NA/Ø therapy/others (A-/allo-SCT)		*33%/30%/37%/n.a*	Comparable
Median # of therapy lines (range)	1 (0–14)	1 (0–10)	Comparable
Radiotherapy: yes:no	32%: 68%	*26%: 74%*	Comparable
*TB-specific data*			
Number of TB presentations: 1x/2x/ ≥ 3x	73%/20%/7%	*58%/25%/17%*	Current pts presented less often 2/ ≥ 3x
Reasons for TB presentations	Therapy: 80%, Staging/ID/diagnosis finding: 20%	*Therapy: 90%,* *Staging/ID/SAE/comorbidities: 9%, other: 1%*	Therapy discussed in 80–90%—> best possible therapy remains main reason for TB presentation
*TB-adherence/evidence levels and pathway-concurrent recommendation/documentation*			
Adherence: TB recommendation followed: yes:no	93%: 7%	*94%: 6%*	Remains > 90%
Reason for non-adherence:			Low non-adherence rate, in all with appropriate reasons^2^
Pt non-consent/Ø implementable/externally impossible^2^	40%/50%/10%	*50%/46%/4%*	
Pathway-concurrent recommendation^3^: yes:no	97%: 3%	*n.a*	> 95% pathway-concordant
Reason why UKF MM-pathway was not pursued^3^:			
Pt non-consent/externally impossible^2^	80%/20%	*n.a*	Low non-concurrent pathway rate
Level of evidence of TB recommendation^4^:			
1A/1B/2A/2B/1C/2C	82%/7%/11%	*77%/19.5%/3.5%*	
TB mentioned in medical reports			
Yes, discussed in detail/yes, included/no	83%/14%/3%	*n.a*	Included in medical reports: 97%

*n* sample size, *n.a.* not assessed, *TB* tumor board, *NA* novel agent, *SCT/ASCT/allo-SCT* stem cell transplantation/autologous/allogeneic tandem-SZT, *Ø* not,—not collected in analysis, *ID* initial diagnosis, *SAE* severe adverse events, + */ − *with or without, *UKF* Universitätsklinikum Freiburg

^1 ^At the time of first MM-TB during evaluation period

^2 ^See Supplement Table 1 for details

^3 ^According to annually updated MM pathway of the UKF/Comprehensive Cancer Center Freiburg and published in: “Blaues Buch,” Springer, 7. Edition; 2020

^4 ^Dimopoulos M. et al. Multiple Myeloma: EHA-ESMO Clinical Practice Guidelines for Diagnosis, Treatment and Follow-up. HemaSphere 5 (2), e528; 2021

### TB adherence, evidence levels, pathway concurrent recommendation, and documentation

Therapy-specific data, TB adherence rates, and evidence levels of decisions were assessed, and it was evaluated if pathway-concurrent recommendations were given and followed. We also determined whether MM-TB recommendations were included in physicians’ reports and, if included, whether these were discussed in detail or remained unmentioned in medical reports (Table 2).

TB adherence to the recommendation was recorded by matching all available documentation data. Grades of recommendations were assigned using the GRADE criteria: *Grade 1* evidence strongly suggests that the benefit of the procedure outweighs potential risks. *Grade 2* evidence suggests that the benefit and risk of a procedure are finely balanced or uncertain. *Grade A* illustrates evidence from systemic reviews of high-quality randomized studies, *B* from randomized and observational studies with methodological flaws, and *C* from randomized and observational studies with major methodological flaws or other sources of evidence (e.g., case series) [1, 9]. All parameters presented in Tables 1 and 2 were compared to our prior data [1]. The comprehensibility or deviation of TB recommendations or MM pathway was reassessed by two MM specialists (ME + RW) according to international MM consensus guidelines (IMWG-, EMN-, Oncopedia-, S3-guidelines, and CCCF MM-pathway) [20]. The CCCF MM-pathway is annually updated by the authors’ team.

### Statistical analyses

OS was defined as the time from first diagnosis to death from any cause and PFS as the time from first diagnosis to cancer recurrence or death from any cause. Data for patients alive at the time of the analysis were censored at the last follow-up. Probabilities of PFS and OS were estimated with Cox regression models accounting for left truncation. This means that patients come under observation at some time later than their ID, namely at the date of the TB presentation. This “late entry” analysis for both PFS and OS was performed to adjust for any selection bias due to longer latencies between the ID and TB presentations, as described [1, 21].

A *p*-value of < 0.05 was considered as statistically significant. Data were analyzed with SAS 9.2 (SAS Institute, Inc, Cary, NC). The study was performed according to the Declaration of Helsinki and Good Clinical Practice. All patients gave their written informed consent for institutionally initiated research studies and analyses of clinical outcome studies conforming to the institutional review board guidelines. The analysis was approved by the UKF ethics committee (EK-EV 20/15).

## Results

### Number of MM-TBs and MM patient characteristics

From March 2020 to February 2021, 312 chronologically discussed MM patients were analyzed in 439 TB protocols, which impressively reflect the substantial assignment of TBs. The median number of TB presentations per patient was one (range: 1–10). The median patient age was 67 years and typical for MM patients at CCCs and referral centers. This was likewise representative in terms of gender, number of comorbidities, performance status (KPS), R-MCI, CCI, and MM-specific characteristics as compared to former analyses of our and other groups [1, 9, 22]. MM patients were discussed within the MM-TB either at ID, at disease recurrence (progressive disease (PD)), or with stable disease (SD) but with the need for additional discussions in 39%, 28%, and 33%, respectively (Table 1).

As displayed in Table 2, reasons for the MM-TB presentation were therapeutic challenges in 80% and questions concerning staging and/or definition of ID in 20%. The numbers of presentations during the 2020/2021 analysis were mostly one in 73%, two in 20%, and three or more in only 7%. Table 2 also summarizes TB-specific data, namely that the medical therapy lines of patients being discussed 2020/2021 were one (range: 0–14). Prior anti-MM therapy of novel agent-based combination treatment alone was performed in 23%, autologous stem cell transplantation (ASCT) in 30%, no therapy being initiated by the time of TB discussion in 41% (including patients with ID), and others in 6% (tandem ASCT/allogeneic [allo]-SCT). The 41% of patients, where the antimyeloma therapy was not intiated by the time of the interdisciplinary TB-discussion, reflected the significance and reliance on the MM-TB, as described similarly for other TBs today. Radiotherapy was performed in 32%.

### TB adherence, TB- and pathway-compliant decisions, and level of evidence

The TB adherence rate was 93%. Reasons for non-adherence were predominantly related to patients’ decisions or reflected challenging inclusion criteria for clinical trials (i.e., narrow inclusion and broad exclusion criteria, desire for treatment close to home).

In line, 97% of the decisions were pathway-compliant, making the annually required CCCF updates and our provision of these worthwhile, i.e., via “Blaues or Rotes Buch” [23, 24], in electronic media, through physicians’ training and through our annual UKF-MM workshop. Non-CCCF-pathway-concordant decisions were analogous to those of the TB — in all coherent (Table 2).

The detailed reanalysis of three patients through two experts (BF + ME), where the TB advice was differently pursued in referral (private) practices (*n* = 3, reflecting 10% of TB-non-adherence; Table 2), was evaluated further (Suppl. Table 1). The other TB-non-adherence (7%) were either patients’ non-consent in 40% and/or if inclusion critieria did not allow clinical trial inclusion in 50% (Table 2). The lesson from the three patients, who were deemed to have been TB-non-concordantly treated, showed that the few modifications were plausible, leading to similar anti-MM therapies. All modifications were performed in referral practices outside the CCCF, either due to as yet unlicensed MM therapies (where insurance approval could have been obtained via TB protocol) or different patient preference (Suppl. Table 1).

To capture TB decisions also by their level of evidence, these were analyzed, grouped by highest (defined as grade 1A/1B), medium (grade 2A/2B), and lowest evidence (grade 1C/2C). Notably, rewarding levels of evidence were obtained with 83%, 14%, and 3%, respectively (Table 2).

### Comparative analysis of this (March 2020–February 2021) and previous TB analysis (June 2012–June 2014) [1]

While the current evaluation covered 12 months (March 2020–February 2021), the former MM-TB evaluation had covered 2 years (2012–2014; founding of the MM-TB in 2012). Of interest, both periods enclosed similar patient numbers and TB protocols with 312 and 299 patients and 439 and 498 TB protocols in 2020/2021 vs. 2012–2014, respectively (Table 1). This reflects the substantial task of TBs, with a marked increase in our 1- vs. 2-year assessment periods.

The median number of TB presentations per patient was one (range: 1–10) in 2020/2021 and two (range: 1–8) in 2012–2014. The gradual increase in TB patients is depicted in Suppl. Fig. 1A and 1B (in Suppl. Fig. 1B with more detailed TB results for 2019 to 2021).

With these increases, a higher time efficiency, including those of TB decisions, is warranted. This can be achieved as illustrated in Tables 1 and 2, as median TB protocols per patient declined. The number of one, two, or three or more presentations during this analysis period was 73%, 20%, and 7% vs. 58%, 25%, 17% in our former study [1], respectively, verifying our opinion on frequent TBs: Therein, we had described that the actual number of repeatedly performed TBs does not induce a survival benefit per se, but was missleadingly achieved due to an imortal time bias [25], which needs to be corrected for [1, 9].

Patient-, myeloma- and therapy-specific data of both 2020/21 and 2012/14 analyses were comparable: standard therapy without ASCT, ASCT, no therapy/pending first-line therapy and tandem ASCT/allo-SCT were performed in 23% vs. 30%, 30% vs. 33%, 41% vs. 37%, and 6% vs. 0%, respectively. Local radiation was performed in 32% vs. 26%, likely due to the today's improved PFS and OS with MM, the longer time span in which patients can receive local treatment and interdisciplinary discussion (Table 2).

The TB adherence was > 90% in both analysis periods. Likewise, high evidence rates and pathway-appropriate procedures were achieved (Table 2).

### Time spent in TBs for preparation and discussion

**Fig. 1:**
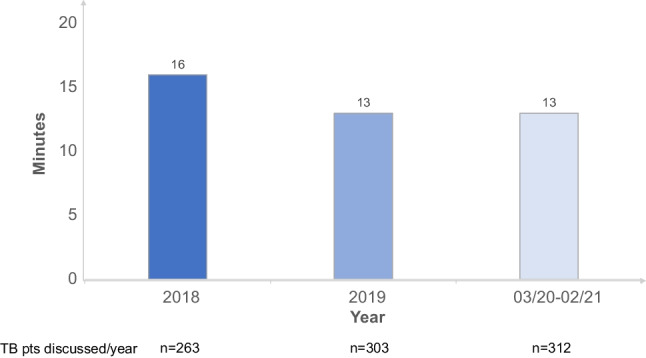
Development of median time requirements for TB participants/pt: 2018 to 2020/2021, which decreased 2018 from 16 min per patient (with *n* = 263 patients being discussed) to 13 min in 2019 (*n* = 303 patients) and again 13 min in 2020/2021 (*n* = 312 patients)

To substantiate a more efficient TB performance, we calculated the time requirements for the preparation, performance, and post-processing (Suppl. Fig. 2A). For 44 TBs in our assessment period March 2020–March 2021 and approximately 30 min estimated preparation time of each participant, this accounted to 1320 min and with 312 patients being discussed, in 4 min/patient/participant. For the TB itself, our calculation accounted for 8 min/patient/participant and for the post-processing time after the TB for 1 min, adding up to 13 min in 2020/2021 (Fig. 1). The comparative calculations for the TBs in 2019 and 2018 are displayed in Suppl. Fig. 2B, which added up to 13 and 16 min, respectively. The median time requirements for TB participants in 2018, 2019, and 2020/2021 are summarized in Fig. 1, where with 16, 13, and 13 min per patient and participants, the time requirements decreased, while TB patients increased from 263 to 303 and 312, respectively.

### Coverage ratio of all MM patients seen and discussed in TB, study inclusion, and referral responses

The German Cancer Society (DKG) measurement tool for the quality of TBs is the “coverage ratio.” This is the proportion of all MM patients being discussed in TBs to those being seen and treated at the CCC (target percentage being > 90%). This yielded a coverage ratio of ID MM patients discussed in the TB in 2020 of 93%, which was 8% higher than in 2013 [1]. The study inclusion rate was 43%. Referral physicians’ satisfaction was anonymously queried via CCCF questionnaire (as previously shown and discussed) [1, 9, 22] and generated excellent satisfaction grades for the MM-TB of 1 (school grades: 1 = very good, 6 = dismal).

### PFS and OS of all patients and via late entry methodology

**Fig. 2:**
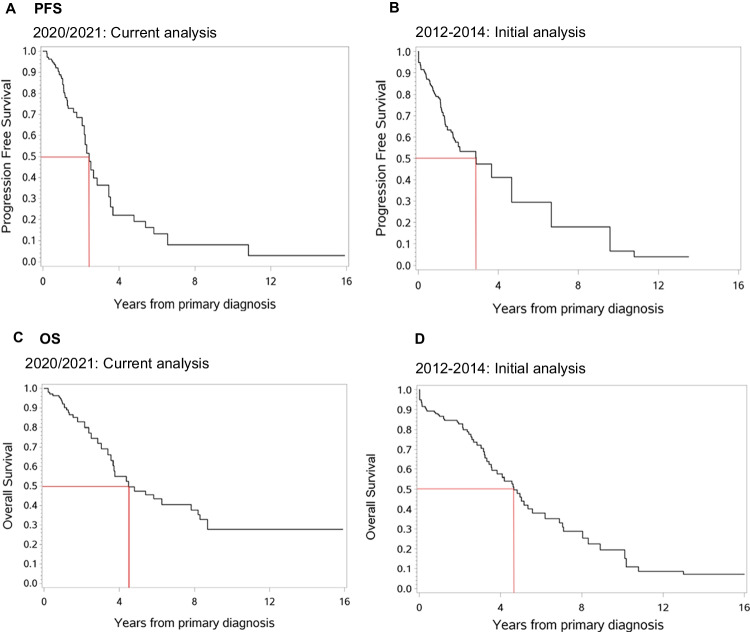
**A**, **B** Comparison of PFS via late-entry method showing comparable results in the current (**A**) as compared to our prior analysis period (**B**). **C**, **D** Comparison of OS via late-entry method showing comparable results in the current (**C**) as compared to our prior analysis period (**D**)

To account for the time lag until patients were discussed in the TB, PFS and OS rates were estimated accounting for left truncation, denoted as “late entry.” The PFS and OS in our 2020/2021 patients vs. prior analysis of 2012–2014 is shown side-by-side in Fig. 2A–D. The 3-year-PFS rate in this current vs. former analysis was 36% and 47%, respectively (Fig. 2A + B). Possible reasons are the shorter analysis period (12 vs. 25 months) and more challenging cases in the current analysis. The 3-year-OS rate was 72% in both cases (Fig. 2C + D).

## Discussion

Although we and others have demonstrated that with the initiation of TBs [1–5], patients discussed therein can be substantially increased, that TB questions mostly involve advice on best treatment, and that levels of compliance and evidence can be as high as > 90% [1]; TBs are resource-intensive. Additional advantages of TBs are that they may improve inclusion into clinical trials and advance interdisciplinary projects [1, 6, 26]. The MM-TB at our CCCF was established in June 2012 and takes place weekly with the discussion of 10–15 patients over a 1-h period per session [1, 9]. The DKG guidelines require the presentation of all cancer patients at ID, with recurrence of the disease and any change or adaptation of diagnostic or therapeutic issues. Due to a constructive, collegial discussion culture to determine the best possible strategy for a patient, hierarchical decision-making in case of disagreement is successfully avoided. In this validation analysis, we demonstrate a substantial increase in patient numbers and MM-TB protocols over a 1- as compared to our former 2-year period [1]. We generated comparable results in challenging to treat MM patients, a high adherence rate to TB recommendations, and pathway-concurrent advice, with high levels of evidence. Of note, time requirements for the TB decreased and PFS and OS were similar. Given that improved PFS and OS via TB advice is specifically challenging to verify (as randomized TB trials cannot be performed for ethical reasons), our validation analysis seems valuable. Additionally, TB patients are often difficult-to-treat and are referred to academic centers due their complexity, therefore questioning comparative analysis in their validity with prior non-TB patients or inaccurate matched-pair analyses. Our survival estimation used the ID as the starting point to provide comparability with other analyses, and the time aspect of TB was considered in the statistical calculations to avoid overestimation of survival probabilities. This statistical refinement and exclusion of an immortal time bias are essential and have been identified as the appropriate statistical approach [1, 12, 21].

The frequency of one, two, or three and more presentations during the analysis period was 73%, 20%, and 7% vs. 58%, 25%, and 17% in our former analysis [1], respectively, verifying our critical opinion on frequent TBs [9]. Of note, this was a 1-year assessment of MM patients being presented and discussed in our MM-TB 2020/2021, not the essence of the entire MM-TB which we started in 2012 (now 10 years ago). Since many MM patients may indeed need multiple lines of therapy during their disease course and may present several times in the MM-TB, this could only have be captured, if a much longer MM-TB “snapshot” had been assessed, i.e., within a 10-year period, since MM patients may survive for 10 years or longer today. Here we assessed a defined time period of 1 year, in which many patients had already been discussed more than once in past MM-TBs, but within our 1-year period again only once (73%), few twice (20%), and very few ≥ 3 times (7%). If we had analyzed all patients since 2012, having now > 400 MM-TB cases/year with > 300 MM patients, this would have accounted for > 4000 MM-TB recommendations and > 3000 MM patients, which would have been a much larger endeavor than that performed with our prior 2-year [1] and now confirmatory 1-year assessment, in both comparing almost exact number of TB presentations and patients to entirely match them in a validation analysis. This led to the “snap shot” distribution of one median MM-TB presentation/year in our patients, which speaks for the durability of our MM-TB advice, the long remission-enduring MM treatment today, and the effectiveness of well-established TBs.

Thus, our comparison of 2020/2021 to 2012–2014 data seemed valuable, because we had described the postulated OS benefit in patients with three or more TB discussions as error-prone, occurring due to an immortal time bias. Here patients need to survive long enough to be discussed more often. Therefore, time-biased results should not lead to the conclusion that more TBs will increase patients’ OS, rather than that the insightful discussion, at best in interdisciplinary teams, will generate meaningful results, that are important for cancer patients [25]. Our focus was therefore — other than in TB analyses with numerous tumor entities — the detailed survey of MM alone. A disadvantage in the comparison of various tumors can be a different approach towards the use of TB or even a standardized algorithm defining the time point and therefore the frequency of discussion within a TB between tumor entities. While one entity might be curable through a defined treatment pathway or surgical intervention without the need to further discuss treatment options, another may not be easily curable [1, 27–29]. These entities require an interdisciplinary discussion in varying frequencies, therefore confounding a possible comparability regarding OS, which is circumvented when focus on one tumor entity is accomplished, as performed here.

Of interest in our preliminary analysis was that tele/ZOOM conferencing and participation therein were not unconditionally supported by referring physicians [1]. Reasons for this were that TBs would be difficult to incorporate into their daily routine in often private outpatient practice, and thus, direct TB-online-system (TOS) access and timely provision of TB protocols were the more viable solution for referring physicians. Overall, our previous surveys of referring physicians to our CCCF confirmed that the close and prompt interaction is crucial to maintain their satisfaction at a high level [1, 9, 22]. Nevertheless, the prolonged SARS-CoV-2 pandemic has implemented an electronic access to all 26 UKF-TBs now, which is eagerly used by participating TB physicians. This allows disciplines with frequent involvement to resourcefully participate and, therein, ensures “distancing” and reliable TB occurrence.

In conclusion, TBs, with the goal of recommending individualized and optimized cancer care, are required as a contemporary standard both in the “National cancer plan of Germany” and by specific guidelines of professional societies [1, 9, 20]. In order to ensure the implementation of TB recommendations in a timely manner, they are immediately available at our electronic TOS and integrated in physician reports. The reliable integration of TB recommendations in physicians’ reports/letters was verified in this validation study in 97%. Albeit previous analyses of TBs, mostly in solid tumor entities, the procedures and results presented here may be useful for other CCCs in each developing their own standards in this area. The parameters studied could be implemented in the context of certification-relevant metrics for TBs to further improve the quality of oncology centers. Further improvements of our TOS is — evolving from our prior analyses [1, 9] — that certification-relevant data can be taken directly from TOS. TOS data is directly integrated into the database of the tumor documentation system ONKOSTAR and easy to link with structured data of clinical cancer registries (i.e., diagnoses, therapy courses, and follow-up) for further analysis. Thus, an electronic consolidation of certification-relevant data takes place, which contributes to the improvement of the presentation for recertifications, therapy recommendations, makes the work of interdisciplinary teams more effective, and more expeditiously ensures the automatic data acquisition for certification processes. Most importantly, these and other important TB analyses have led to our sounder interpretation of cancer care, in close collaboration with statisticians [1, 8, 12, 21, 22], which is essential to produce reliable evidence for future progress. We are gratified that many productive collaborations continue to exist at our and other CCCs.

## Supplementary Information

Below is the link to the electronic supplementary material.

Supplementary file1 (DOCX 18 KB)

Supplementary file2 (PPTX 61 KB) **Supplement Figure 1A.** Development of number of patients discussed in our MM-TB during the last 10 years, showing a gradual but continous increase in patients. **Supplement Figure 2A. **Our calculation of the time required per TB participant confirmed the assumption of swifter TB-performance. For 44 TBs in our assessment period 3/2020-3/2021 and approximately 30 minutes estimated preparation time for each participant, this accounted to 1320 minutes and with 312 patients being discussed, in 4 minutes/patient/participant. For the TB itself, our calculation accounted for 8 minutes/patient/participant and for the post processing time after the TB for 1 minute, adding up to 13 minutes in 2020/2021. **Supplement Figure 2B.** The comparative calculations for the TBs in 2019 and 2018 are displayed in Suppl. Fig. 2B, which added up to 13 and 16 minutes, respectively. The median time requirements for TB participants in 2018, 2019 and 2020/2021 is summarized in Fig. 1, where with 16, 13 and 13 minutes per patient and participant, the time requirements did decrease, whilst TB patients increased from 263 (2018), to 303 (2019) and 312 (2020/20)
