# Supplementary material for: Multidisciplinary tumor board analysis: validation study of a central tool in tumor centers
Source: Ann Hematol. 2022 Dec 5;102(3):603–11. doi: 10.1007/s00277-022-05051-y (PMC9734518; doi:10.1007/s00277-022-05051-y)
Supplement: Supplementary file 1 — Supplementary file1 (DOCX 18 KB) [file 277_2022_5051_MOESM1_ESM.docx]

**Supplement Table 1. MM-TB-divergence from MM-TB-recommendations**

**(n=3; 10% with adapted treatment performed in private practice [PP])**

| **Variables** | **Patient #1** | **Patient #2** | **Patient #3** |
| --- | --- | --- | --- |
| ***MM-type, ID*** | Kappa-LC MM, 4/2021 | IgG kappa, 4/2017 | Kappa LC MM; 5/2013 |
| ***Patient age at ID (years)*** | 58 | 67 | 43 |
| ***Gender*** | female | male | male |
| ***Revised-Myeloma Comorbidity Index*** | 0/9 = fit | 6/9 = intermediate-fit | 4/9 = intermediate-fit |
| ***LT discussed in MM-TB*** | 1.LT | 4.LT | 6.LT |
| ***MM-TB recommendation*** | VCD + ASCT | Due to t(11;14):  Venetoclax (Ven)-Cfz (K)-Dex (Ven-Kd) | Belantamab-Pom-Dex according to ASH_2020+2021_,  alternatively Isa-Pd or Selinexor-Vd |
| ***Treatment performed in PP*** | Dara-Rd | K-Cyclo-Dex (KCd) | Kd -> K-Benda-Dex |
| ***Reason, why MM-TB recommendation was modified in PP*** | - Anxious, depressed pt - Pt initially declined to receive ASCT | - Ven-Kd not licensed   albeit MM-TB recommendation eases approval | - Belantamab+Pom not licensed albeit MM-TB recommendation eases approval |
| ***Outcome*** | After 6 cycles Dara-Rd: ASCT | PD on KCd | PD on Kd + K-Benda-Dex |
| ***Last remission status Alive / died*** | VGPR  Alive | PD  Died 11/2020 | PD  Died 5/2021 |
| ***Case lesson to be learned*** | - MM-TB advice followed with ASCT | - KCd used, no Ven-combo due to unlicensed status - Targeted approach for t(11;14) remained unused | - K-combinations used 2x unsuccessfully - BCMA-ADC approach with Belantamab remained unused |
| ***Summary of all 3 pts*** | - Few modifications, all plausible & reproducible, leading to same or similar therapies. - All modifications performed in PP due to  1. pt preference and 2. as yet unlicensed MM-therapies, albeit insurance approval could have been obtained via MM-TB protocols. | | |

**Abbreviations:**

MM: multiple myeloma, MM-TB: MM-tumor board, PP: private practice, #: number, pt/pts: patient / patients, LC: light chain only multiple myeloma (MM), LT: therapy line, PD: progressive disease,

VCD: bortezomib-cyclophosphamide-dexamethasone, Cfz-Cyclo-Dex: Carfilzomib-Cyclophsophamide-Dexamethason (KCd), Dara-Rd: Daratumumab-lenalidomide-dexamethasone according to Maia study, Ven-combo: venetoclax combination, Pom: pomalidomide, Isa-Pd: Isatuximab-pomalidomide,dexamethasone, Selinexor-Vd: Selinexor-bortezomib-dexamethasone, K: Carfilzomib, K-Benda-Dex: Carfilzomib-Bendamustin-Dexamethasone.
