# Supplementary material for: Multidisciplinary tumor board analysis: validation study of a central tool in tumor centers
Source: Ann Hematol. 2022 Dec 5;102(3):603–11. doi: 10.1007/s00277-022-05051-y (PMC9734518; doi:10.1007/s00277-022-05051-y)
Supplement: Supplementary file 2 — Supplementary file2 (PPTX 61 KB) Supplement Figure 1A. Development of number of patients discussed in our MM-TB during the last 10 years, showing a gradual but continous increase in patients. Supplement Figure 2A. Our calculation of the time required per TB participant confirmed the assumption of swifter TB-performance. For 44 TBs in our assessment period 3/2020-3/2021 and approximately 30 minutes estimated preparation time for each participant, this accounted to 1320 minutes and with 312 patients being discussed, in 4 minutes/patient/participant. For the TB itself, our calculation accounted for 8 minutes/patient/participant and for the post processing time after the TB for 1 minute, adding up to 13 minutes in 2020/2021. Supplement Figure 2B. The comparative calculations for the TBs in 2019 and 2018 are displayed in Suppl. Fig. 2B, which added up to 13 and 16 minutes, respectively. The median time requirements for TB participants in 2018, 2019 and 2020/2021 is summarized in Fig. 1, where with 16, 13 and 13 minutes per patient and participant, the time requirements did decrease, whilst TB patients increased from 263 (2018), to 303 (2019) and 312 (2020/20) [file 277_2022_5051_MOESM2_ESM.pptx]

## Slide 1
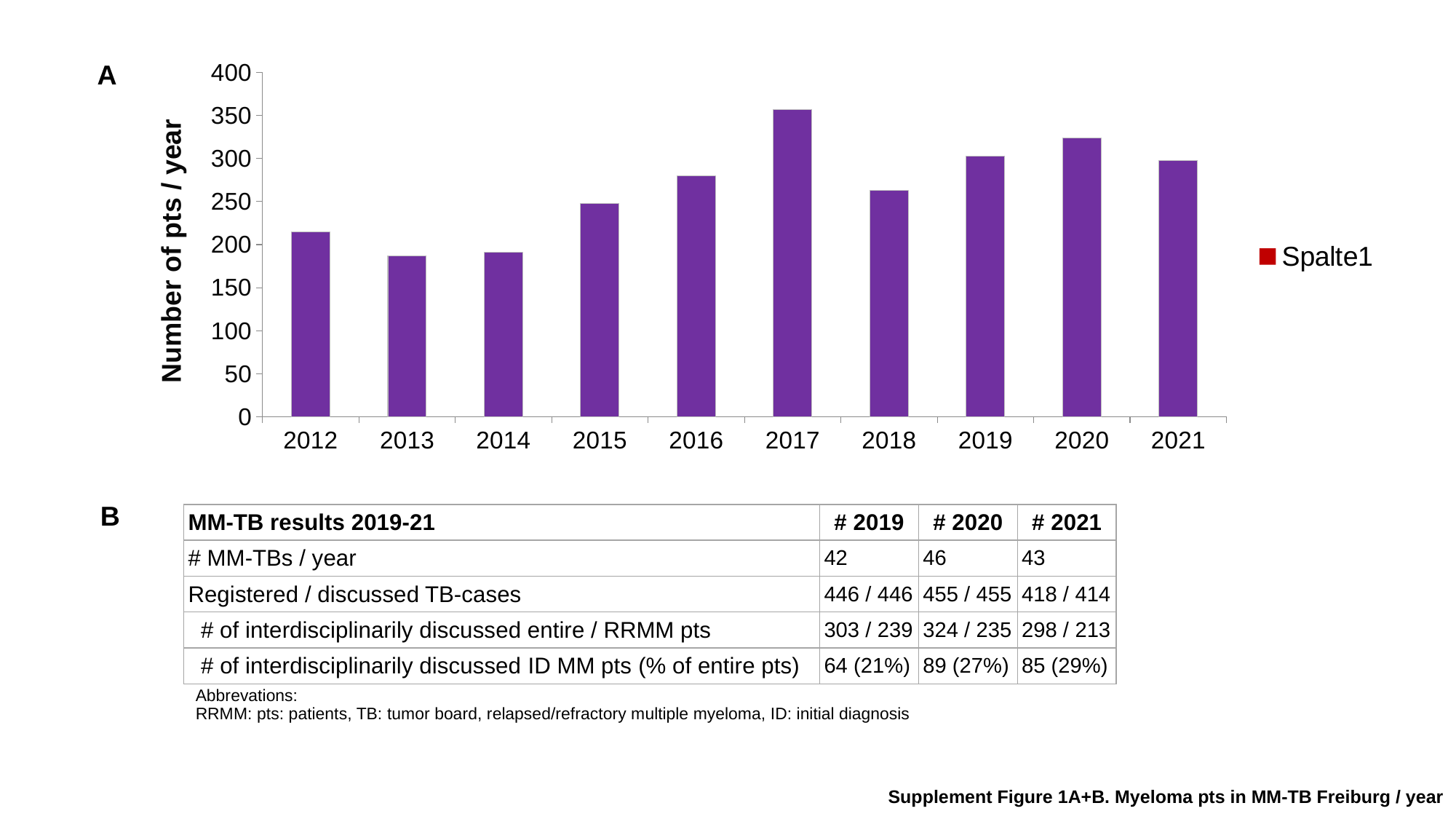

### Chart
| Category | # of discussed pts | Spalte1 |
|---|---|---|
| 2012 | 215.0 | None |
| 2013 | 187.0 | None |
| 2014 | 191.0 | None |
| 2015 | 248.0 | None |
| 2016 | 280.0 | None |
| 2017 | 357.0 | None |
| 2018 | 263.0 | None |
| 2019 | 303.0 | None |
| 2020 | 324.0 | None |
| 2021 | 298.0 | None |A
Number of pts / year
B
| MM-TB results 2019-21 | # 2019 | # 2020 | # 2021 |
| --- | --- | --- | --- |
| # MM-TBs / year | 42 | 46 | 43 |
| Registered / discussed TB-cases | 446 / 446 | 455 / 455 | 418 / 414 |
| # of interdisciplinarily discussed entire / RRMM pts | 303 / 239 | 324 / 235 | 298 / 213 |
| # of interdisciplinarily discussed ID MM pts (% of entire pts) | 64 (21%) | 89 (27%) | 85 (29%) |
Abbrevations:
RRMM: pts: patients, TB: tumor board, relapsed/refractory multiple myeloma, ID: initial diagnosis
Supplement Figure 1A+B. Myeloma pts in MM-TB Freiburg / year

## Slide 2
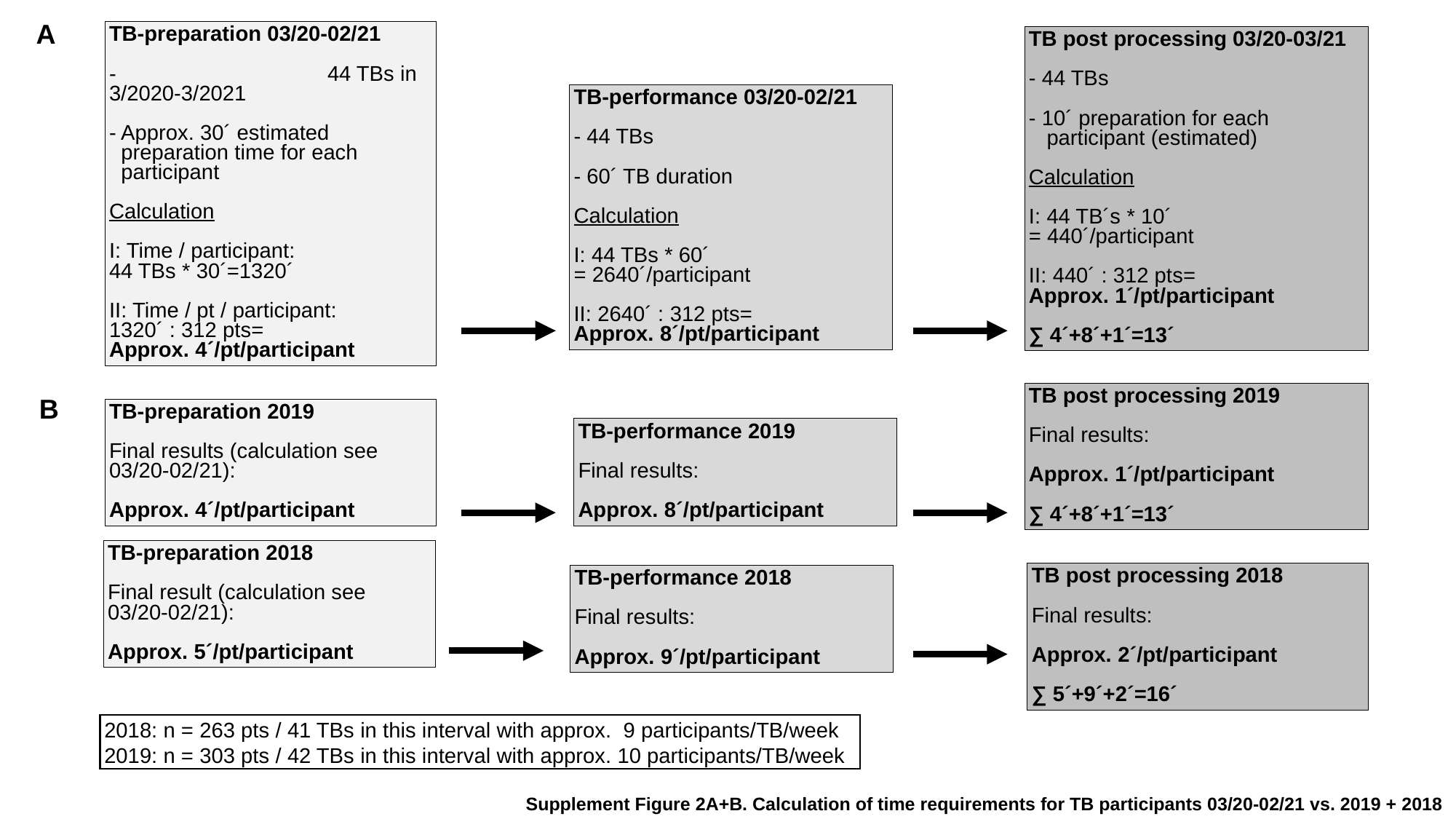

A
TB-preparation 03/20-02/21
-		44 TBs in 3/2020-3/2021
- Approx. 30´ estimated
 preparation time for each
 participant
Calculation
I: Time / participant:
44 TBs * 30´=1320´
II: Time / pt / participant:
1320´ : 312 pts=
Approx. 4´/pt/participant
TB post processing 03/20-03/21
- 44 TBs
- 10´ preparation for each
 participant (estimated)
Calculation
I: 44 TB´s * 10´
= 440´/participant
II: 440´ : 312 pts=
Approx. 1´/pt/participant
∑ 4´+8´+1´=13´
TB-performance 03/20-02/21
- 44 TBs
- 60´ TB duration
Calculation
I: 44 TBs * 60´
= 2640´/participant
II: 2640´ : 312 pts=
Approx. 8´/pt/participant
TB post processing 2019
Final results:
Approx. 1´/pt/participant
∑ 4´+8´+1´=13´
B
TB-preparation 2019
Final results (calculation see 03/20-02/21):
Approx. 4´/pt/participant
TB-performance 2019
Final results:
Approx. 8´/pt/participant
TB-preparation 2018
Final result (calculation see 03/20-02/21):
Approx. 5´/pt/participant
TB post processing 2018
Final results:
Approx. 2´/pt/participant
∑ 5´+9´+2´=16´
TB-performance 2018
Final results:
Approx. 9´/pt/participant
2018: n = 263 pts / 41 TBs in this interval with approx. 9 participants/TB/week
2019: n = 303 pts / 42 TBs in this interval with approx. 10 participants/TB/week
# Supplement Figure 2A+B. Calculation of time requirements for TB participants 03/20-02/21 vs. 2019 + 2018
